# Vaccinia virus protein A49 activates Wnt signalling by targetting the E3 ligase β-TrCP

**DOI:** 10.1099/jgv.0.000946

**Published:** 2017-10-17

**Authors:** Carlos Maluquer de Motes, Geoffrey L. Smith

**Affiliations:** Department of Pathology, University of Cambridge, Tennis Court Road, CB2 1QP, Cambridge, UK; ^†^​ Present address: Department of Microbial Sciences, University of Surrey, GU2 7XH, Guildford, UK.

**Keywords:** vaccinia virus, protein A49, β-transducin repeat containing protein, Wnt pathway, β-catenin

## Abstract

Vaccinia virus (VACV) encodes multiple proteins inhibiting the NF-κB signalling pathway. One of these, A49, targets the E3 ubiquitin ligase β-TrCP, which is responsible for the ubiquitylation and consequential proteosomal degradation of IκBα and the release of the NF-κB heterodimer. β-TrCP is a pleiotropic enzyme ubiquitylating multiple cellular substrates, including the transcriptional activator β-catenin. Here we demonstrate that A49 can activate the Wnt signalling pathway, a critical pathway that is involved in cell cycle and cell differentiation, and is controlled by β-catenin. The data presented show that the expression of A49 ectopically or during VACV infection causes accumulation of β-catenin, and that A49 triggering of Wnt signalling is dependent on binding β-TrCP. This is consistent with A49 blocking the ability of β-TrCP to recognise β-catenin and IκBα, and possibly other cellular targets. Thus, A49 targetting of β-TrCP affects multiple cellular pathways, including the NF-κB and Wnt signalling cascades.

Vaccinia virus (VACV), the prototypic member of the *Poxviridae*, encodes many proteins that interfere with the host immune response and thereby generate a cellular environment that is conducive for viral replication and spread. Some of these factors act extracellularly and bind to cytokines and chemokines that are critical for immune activation and the recruitment of immune cells to the site of infection [1]. Some others act intracellularly and inhibit signalling cascades leading to the production of those cytokines and chemokines, or manipulate cellular pathways to facilitate viral protein synthesis, or prevent premature cell death and hence abortion of the viral replication cycle [2, 3]. In some cases, a single protein functions in multiple ways and each function can be ascribed to discrete parts of the viral protein. For instance, protein C16 targets the oxygen-sensing enzyme PHD-2 and induces a hypoxic response that is metabolically beneficial for the virus [4, 5], but also inhibits inflammatory signalling deriving from cytosolic DNA sensing [6]. Protein N1 also blocks inflammatory signalling downstream of several receptors, but also prevents cell death [7–10]. This is also true for protein F1 [11–14], although via a different mechanism [15, 16]. This extensive functional redundancy implies that during infection the concerted action of all viral proteins ensures a rapid and efficient manipulation of cellular functions.

Previously, we reported that VACV protein A49 is a virulence factor and inhibits activation of the pro-inflammatory transcription factor NF-κB [17]. A49 is a small protein that structurally resembles members of the cellular B-cell lymphoma (Bcl)−2 family [18], a structural fold that is shared by a number of VACV proteins [19]. In addition to the Bcl-2 core, A49 contains an unstructured N-terminal extension containing a motif resembling that recognised by the cellular E3 ubiquitin ligase β-TrCP (also known as FBXW11). β-TrCP is the enzyme responsible for the ubiquitylation and degradation of the inhibitor of κB (IκB)α, the molecule that retains the NF-κB heterodimer in the cell cytosol [20]. β-TrCP recognises the specific sequence Ser–Gly–Asn–Leu–Ser in IκBα when the two Ser residues are phosphorylated (Ser 32/36) and conjugates ubiquitin to upstream lysine residues, leading to the proteasomal degradation of IκBα [21, 22]. The A49 N-terminal extension mimics the IκBα sequence recognised by β-TrCP, including the double serine, but not the ubiquitin acceptor lysines upstream of it. As such, A49 binds β-TrCP without compromising its own stability, but blocks β-TrCP recognition of IκBα, and hence NF-κB activation.

The cellular roles for β-TrCP extend beyond NF-κB activation. The β-TrCP recognition sequence, or degron, is present in multiple cellular proteins, and β-TrCP regulates several cellular processes [23]. Targetting of β-TrCP therefore has the potential to affect multiple cellular pathways. To determine whether A49 behaves as a broad β-TrCP inhibitor, or rather displays certain specificity towards the β-TrCP control of NF-κB, the effects of A49 on another cellular pathway controlled by β-TrCP, the Wnt pathway, were explored. The Wnt signalling pathway is important in cell differentiation and development and becomes active after the accumulation of cytosolic β-catenin and its subsequent translocation to the nucleus [24]. In resting cells, β-catenin levels remain low due to its constitutive phosphorylation by glycogen synthase kinase (GSK)−3β and subsequent ubiquitylation by β-TrCP and degradation. Thus β-TrCP controls β-catenin levels and prevents activation of Wnt signalling, and so targetting of β-TrCP by A49 could reverse this.

To determine the effect of A49 targetting of β-TrCP on β-catenin accumulation and Wnt activity, THP-1 monocytic cells were infected at 10 p.f.u./cell with VACV Western Reserve lacking A49 (vΔA49) and its revertant control (vA49rev) in which the A49 gene was reinserted in its natural locus [17]. The levels of β-catenin were monitored over time by immunoblotting using an antibody against β-catenin (Millipore). The levels of β-actin (Sigma-Aldrich) and the viral proteins D8 [25] and A49 [17] were also assessed. Infection with vΔA49 did not affect β-catenin levels and these remained similar to those observed in non-infected cells (Fig. 1a). Conversely, vA49rev infection induced a progressive accumulation of β-catenin over time that correlated with the presence of A49. This experiment was repeated using quantitative fluorescently labelled secondary antibodies (LI-COR), and β-catenin levels were normalised to those of viral protein D8. When these ratios were compared between vΔA49 and vA49rev infections, a statistically significant accumulation of β-catenin was observed at 10 and 24 h p.i. (Fig. 1b). HEK293T cells were then transfected with a plasmid encoding A49 fused to a tandem-affinity purification (TAP) tag containing FLAG epitopes [17] or its corresponding empty vector (EV), and 24 h later cells were treated with medium containing a Wnt3A agonist (a gift from Mariann Bienz, Laboratory of Molecular Biology) for a further 6 h. The cells were lysed and divided into cytosolic and nuclear fractions using a fractionation kit (Pierce), and the β-catenin levels were measured by immunoblotting. Cells transfected with A49 had higher levels of β-catenin in both the cytosolic and nuclear fractions (Fig. 1c). Treatment with Wnt3A triggered β-catenin accumulation, particularly in the cytosolic fraction. In the nucleus, β-catenin accumulation was more apparent when both A49 expression and Wnt3A treatment occurred, which also caused accumulation of nuclear A49. These data demonstrated that A49 is both necessary and sufficient for β-catenin accumulation.

**Fig. 1.**
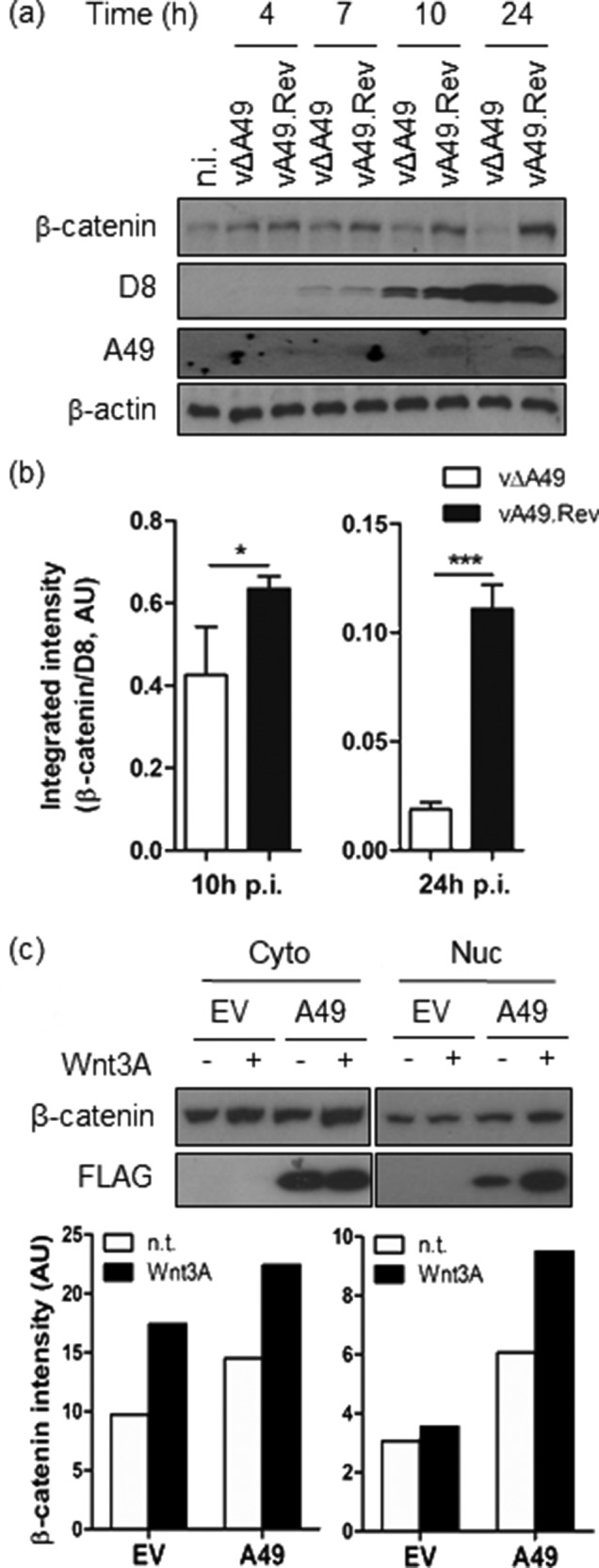
A49 expression leads to β-catenin accumulation. (a) THP-1 cells were infected with VACV lacking A49 (vΔA49) or its revertant control (vA49.Rev) at 10 p.f.u./cell and lysed at different hours (h) post-infection with RIPA buffer. Whole-cell lysates were subjected to immunoblotting as indicated. (b) Levels of β-catenin and viral protein D8 were determined at 10 and 24 h post-infection (p.i.) with vΔA49 (white bars) or vA49.Rev (black bars) from three different experiments, and the corresponding ratios were plotted as the mean ±SD. Statistical analysis was performed using a Student’s *t*-test. *, *P*-value<0.05; ***, *P*-value<0.001. (c) Levels of β-catenin and A49 (FLAG) were determined by immunoblotting in the cytosolic (Cyto) and nuclear (Nuc) fractions of HEK293T cells previously transfected for 24 h with TAP-tagged A49 or the corresponding empty vector (EV), and treated subsequently for 6 h with Wnt3A-containing medium (black bars) or left untreated (white bars). One of two independent experiments with similar results is shown. AU, arbitrary units.

Translocation of β-catenin to the nucleus promotes its binding to TCF/LEF transcription factors and dissociation from transcriptional repressors [24]. To determine whether A49 was capable of triggering Wnt transcriptional activation, a reporter plasmid expressing firefly luciferase under the control of TCF/LEF transcriptional control elements (Promega) was used. HEK293T cells were seeded and transfected as described [17] with a TCF/LEF-Luc reporter, a Renilla luciferase (RLuc) control reporter and either TAP-tagged A49 or its EV control. and then 24 h later the cells were treated with Wnt3A medium for 6 h or left untreated. In EV-transfected cells, the Wnt3A treatment induced a twofold increase in TCF/LEF activity (Fig. 2a). However, in A49-transfected cells a fourfold increase was achieved, and this difference was statistically significant. More interestingly, A49 also induced a statistically significant activation in non-stimulated cells, indicating that the presence of A49 alone was sufficient to increase TCF/LEF reporter activity. This Wnt signalling activation experiment was repeated with the co-transfection of a plasmid encoding FLAG-tagged β-catenin instead of Wnt3A-containing medium. In these conditions, the presence of A49 did not induce further TCF/LEF activity (Fig. 2b). This suggested that β-catenin over-expression and accumulation masked any effect mediated by A49. Finally, the effect of A49 on two cell types known to behave differently in terms of Wnt signalling – U2-OS and HCT116 – was investigated. U2-OS cells derive from a human osteosarcoma and respond to Wnt stimulation, whereas HCT116 cells are a human colon cancer cell line with constitutive Wnt activation caused by a mutation in β-catenin that causes its accumulation [26]. Transfection of A49 induced significant Wnt activation (~30-fold) in U2-OS cells (Fig. 2c). In HCT116 cells, despite its higher basal levels of Wnt activity, transfection of A49 did not induce further TCF/LEF activity when compared to EV-transfected cells (Fig. 2c). This is consistent with A49 affecting the Wnt pathway at the level of β-catenin and not downstream of it. Taken together, these data demonstrate that A49 expression triggers β-catenin accumulation and Wnt signalling activation.

**Fig. 2.**
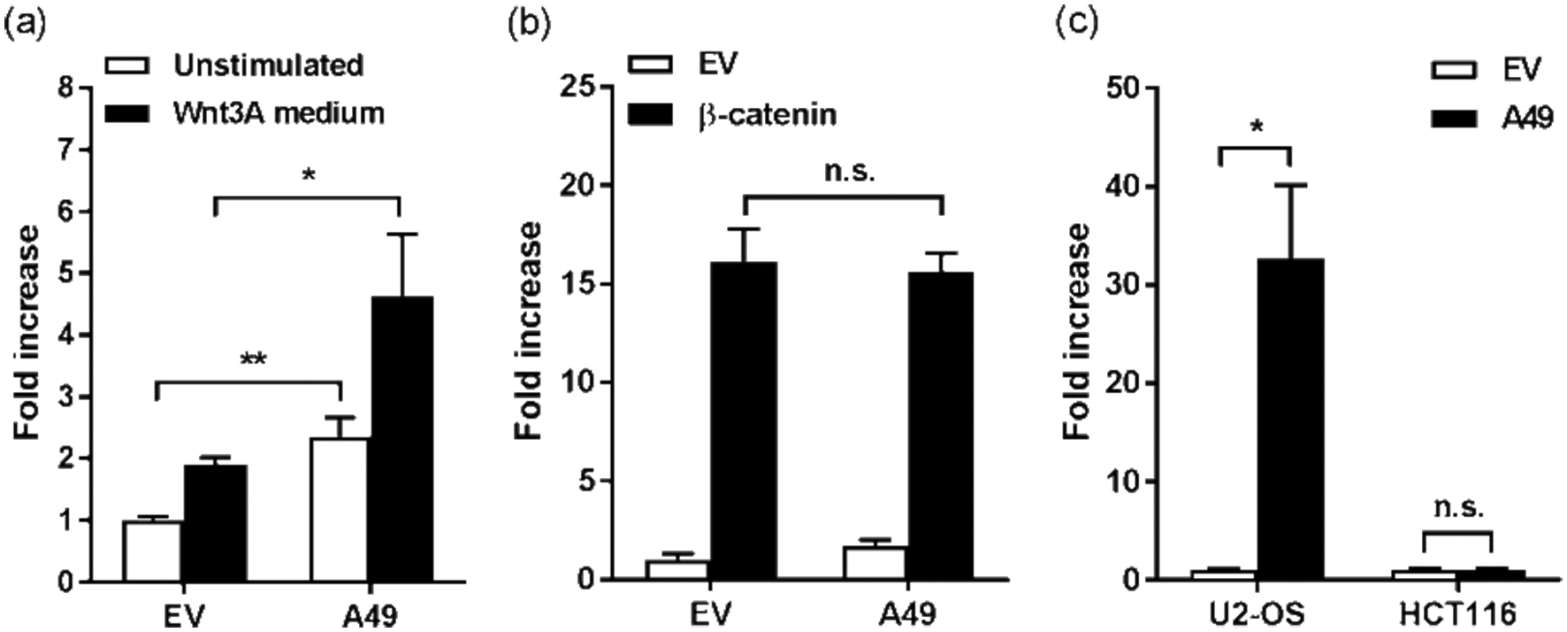
A49 triggers Wnt signalling activity. (a) HEK293T cells were transfected for 24 h with a Wnt-specific luciferase reporter, a control reporter, and TAP-tagged A49 or empty vector (EV). The cells were then stimulated for a further 6 h with Wnt3A-containing medium (black bars) or left unstimulated (white bars). Luciferase activity was calculated and plotted as the mean fold increase ±SD over the unstimulated EV condition. (b) HEK293T cells were transfected as above, as well as with 50 ng per well of a plasmid encoding FLAG-tagged β-catenin (black bars) or EV (white bars). Luciferase activity was calculated 24 h post-transfection as above. (c) U2-OS or HCT116 cells were transfected with TAP-tagged A49 (black bars) or EV (white bars). Luciferase activity was calculated 24 h post-transfection as above. One of at least three independent experiments performed in triplicate is shown in each panel. Statistical analysis was performed using a Student’s *t*-test. *, *P*-value<0.05; **, *P*-value<0.01.

To confirm that the A49 effect on Wnt activation derives from its ability to target β-TrCP, A49 mutants in which the N-terminal extension that contains the β-TrCP recognition motif Ser–Gly–Asn–Leu–Glu–Ser (SGNLES) had been modified were used (Fig. 3a). Firstly, these mutants were assessed for their ability to bind β-TrCP in LUMIER assays [27]. β-TrCP was cloned as a fusion to RLuc and over-expressed in HEK293T cells in conjunction with each TAP-tagged A49 allele. The VACV protein C6 was used as a control [28]. FLAG-tagged Vpu (a gift from Paul Lehner, Cambridge Institute for Medical Research) was also included, because this human immunodeficiency virus (HIV) protein interacts with β-TrCP [29]. Cell lysates were prepared 24 h post transfection and subjected to immunoprecipitation with FLAG agarose (Sigma-Aldrich) as described [8], utilising the FLAG epitope present in all TAP-tagged proteins and Vpu. Rluc activity was measured in both the inputs and the final immunoprecipitated eluates, and a ratio between these activities was calculated for each condition and plotted as a binding fold over the C6 control. As expected, wild-type A49 (A49.WT) associated with β-TrCP and this was quantitated as a ~fivefold increase over control (Fig. 3b). HIV Vpu bound β-TrCP to a higher extent, in agreement with its higher expression level. Conversely, a mutant of A49 lacking the first 12 residues (A49.Δ12), and hence the entire β-TrCP degron, showed no binding, despite higher expression levels than A49.WT. Replacement of the two critical Ser residues for Ala (S7/12A) abrogated binding to β-TrCP, although a minimal but reproducible association was detected and showed statistical significance. In contrast to A49.S/A, substitution of both Ser for Glu (S7/12E) restored and even enhanced binding compared to A49.WT. This indicates that in A49 Glu efficiently mimics phosphorylated Ser, in agreement with previous observations [17]. These mutants were then tested for their ability to activate Wnt signalling (Fig. 3c). As in Fig. 2(a), A49.WT induced a ~twofold increase in reporter activity, and this was enhanced up to ~fourfold after treatment with Wnt3A agonist. Conversely, A49.Δ12 did not activate Wnt signalling. This was also the case for A49.S/A. However, this mutant did trigger statistically significant reporter activity after activation with Wnt3A, possibly reflecting its marginal ability to bind β-TrCP (shown in Fig. 3b). Remarkably, A49.S/E doubled the extent of Wnt activation compared to A49.WT, and this was barely increased by Wnt3A treatment, suggesting that HEK293T cells were unable to respond any further to Wnt signalling stimulation. The levels of A49 from these lysates were assessed by immunoblotting. Whilst the levels of both A49.Δ12 and A49.S/A remained similar, those of A49.WT and A49.S/E were lower when Wnt3A treatment was applied. This observation might be ascribed to the enhanced stabilisation or translocation of A49 in the nucleus upon Wnt3A activation, a phenomenon already reported in Fig. 1c that affects the A49 alleles engaging β-TrCP for reasons that remain unclear. In summary, these data demonstrate that the ability of A49 to target β-TrCP correlates with its ability to activate Wnt signalling, and that this resides in the β-TrCP degron motif located at the N terminus.

**Fig. 3.**
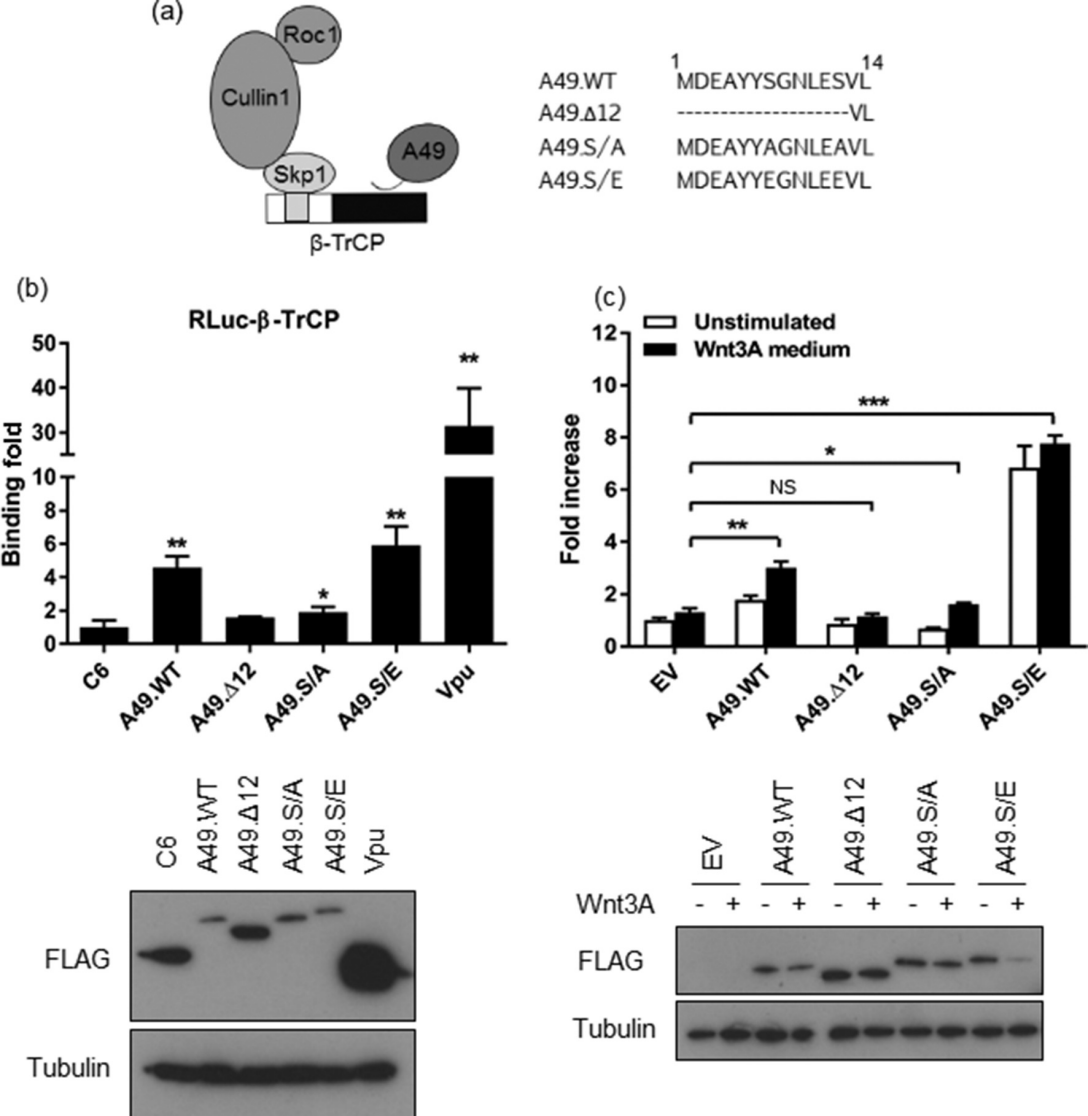
The N-terminal extension of A49 determines its ability to target β-TrCP and to activate Wnt signalling. (a) Schematic of A49 targetting of the E3 ubiquitin ligase complex containing β-TrCP and mutagenesis of the N-terminal extension of A49 in which residues 1–12 were deleted (A49.Δ12), or Ser 7 and 12 residues were changed to Ala (A49.S/A) or Glu (A49.S/E). (b) LUMIER assay showing the ability of each A49 protein to associate with β-TrCP fused to renilla luciferase (RLuc-β-TrCP). HEK293T cells were co-transfected in triplicate with RLuc-β-TrCP and each of the TAP-tagged A49 alleles, C6 or Vpu as indicated, and 24 h later luciferase activity was measured in the cell lysates and also in the eluates deriving from FLAG immunoprecipitation. The ratio lysate/eluate for each condition was calculated and plotted as the mean binding fold ±SD over the C6 control condition. Immunoblotting for FLAG or α–tubulin from the same lysates is shown below, as indicated. One of two experiments performed in triplicate is shown. (c) Luciferase assay showing the ability of each A49 protein to activate Wnt signalling. HEK293T cells were transfected for 24 h with a Wnt-specific luciferase reporter, a control reporter and each of the TAP-tagged A49 alleles or EV, as indicated. The cells were then stimulated for a further 6 h with Wnt3A-containing medium (black bars) or left unstimulated (white bars). Luciferase activity was calculated and plotted as the mean fold increase ±SD over the unstimulated EV condition. Immunoblotting for FLAG or α–tubulin from the same lysates is shown below. Replicate wells were pooled and centrifuged at 14 000 ***g*** for 30 mins, and post-nuclear supernatants were subjected to SDS-PAGE as indicated. One of at least three independent experiments performed in triplicate is shown. Statistical analysis was performed using a Student’s *t*-test. *, *P*-value<0.05; **, *P*-value<0.01; ***, *P*-value<0.001; , not significant.

A49 is one of many VACV proteins that inhibit NF-κB signalling and contribute to virulence. The NF-κB pathway is a critical signalling pathway in inflammation and contributes to type I interferon (IFN) production. Hallmarks of NF-κB activation include the phosphorylation of IκBα by the upstream kinase IκB kinase (IKK)-β; the subsequent degradation of IκBα; and the translocation of the NF-κB heterodimer p65/p50 into the nucleus. Most NF-κB inhibitors encoded by VACV target cellular components required for the extracellular signal to transduce and induce IκBα phosphorylation [2], although there is evidence that VACV inhibits NF-κB activation downstream of p65 translocation [30]. In contrast, and somewhat counterintuitively for an NF-κB inhibitor, protein A49 does not prevent IκBα phosphorylation. It does, however, block NF-κB activation by preventing the degradation of phosphorylated IκBα, a phenomenon explained by its ability to bind and inhibit β-TrCP [17]. β-TrCP is a pleiotropic E3 ubiquitin ligase targetting substrates involved in multiple cellular processes. Consequently, VACV targetting of β-TrCP can dysregulate other cellular pathways in addition to inhibiting NF-κB activation. These data presented here indicate that the VACV protein A49 activates Wnt signalling, and this is dependent on its ability to target β-TrCP. This indicates that VACV targetting of β-TrCP is not specific to NF-κB regulation, and that A49 has not evolved mechanisms to distinguish between β-TrCP molecules involved in NF-κB or Wnt activation, and may consequently act as a broad β-TrCP inhibitor. Another well-described viral interactor of β-TrCP is the HIV protein Vpu. Vpu also binds β-TrCP via a degron sequence that is similar to that found in IκBα or β-catenin [29]. Thus, it shares striking mechanistic similarities with A49. Consistent with the pan-β-TrCP inhibitor model, Vpu also triggered Wnt activation in our assays (data not shown). This suggests that viral proteins harbouring the β-TrCP degron sequence can block the degradation of β-TrCP substrates and interfere with their biological pathways.

A49 targetting of β-TrCP inhibits NF-κB activation, induces Wnt signalling and has the potential to alter other β-TrCP-controlled cellular processes. This raises the following questions: (1) what is the principal function of A49? and (2) is all of the A49-induced activity beneficial for the virus? It is formally very difficult to tease apart the different functions of A49 if they all lie in the β-TrCP degron motif and rely on the interaction with β-TrCP. It is important to note that the interaction between A49 and β-TrCP depends on the phosphorylation of the Ser residues in the degron. We do not currently know which kinase(s) phosphorylate(s) A49, but it is conceivable that this is performed by the same kinases that usually phosphorylate β-TrCP substrates, e.g. IKK-β, which phosphorylates IκBα in the NF-κB pathway, or GSK-3β, which phosphorylates β-catenin in the Wnt pathway [24]. Phosphorylation by IKK-β or GSK-3β would allow A49 to interact with β-TrCP in response to specific cellular challenges, NF-κB or Wnt signalling agonists in this case. Therefore, the post-translational phosphorylation of A49 may be a regulatory mechanism determining the A49 function and activity. Of note and in contrast to most VACV immunomodulators, which are expressed early during infection, A49 is expressed throughout the entire viral cycle, perhaps fulfilling different roles at different stages.

Whether Wnt activation or any other phenotype caused by A49 targetting of β-TrCP is beneficial or detrimental to VACV is currently unknown. Our data indicate that VACV infection leads to β-catenin accumulation, but an increase in Wnt reporter activity when cells were infected with WR at different m.o.i.s was not seen (data not shown). This may indicate that VACV interferes with Wnt signalling downstream of β-catenin through the concerted action of one or more viral proteins. It is also possible that the β-catenin transcriptional activation is masked in our experimental setting by the gross changes induced by VACV infection. Multiple viruses affect Wnt signalling (reviewed in [31]). HHV-5 and HHV-1 cause cellular redistribution of β-catenin [32–34]. Cancer-causing viruses, such as the human herpesvirus (HHV)−4, HHV-8, human papillomavirus, or human polyomavirus JC, upregulate β-catenin and Wnt gene expression. Interestingly, HHV-4 late membrane protein (LMP)−1, which interacts with β-TrCP via an IκB-like degron [35], induces nuclear accumulation of β-catenin [36], suggesting that LMP-1 and A49 activate Wnt signalling by similar mechanisms. VACV and poxviruses in general are cytolytic and do not cause cancer. Manipulation of β-catenin and Wnt signalling may, however, provide optimal cell cycle conditions for virus replication. Interestingly, a role for the Wnt pathway in modulating the host inflammatory and anti-viral response was reported recently [37–44]. Although the outcome of this modulation may differ between experimental settings, in some cases Wnt activity supports viral replication and dampens innate immune signalling [42–44]. This model would complement the role of A49 in down-regulating NF-κB activation and the IFN response. Future studies will establish the contribution of β-catenin accumulation and Wnt signalling to this down-regulation.
